# Balloon Valvuloplasty for Congenital Aortic Stenosis: Experience at a Tertiary Center in a Developing Country

**DOI:** 10.1155/2021/6681693

**Published:** 2021-01-12

**Authors:** Fatme A. Charafeddine, Haytham Bou Houssein, Nadine B. Kibbi, Issam M. El-Rassi, Anas M. Tabbakh, Mohammad S. Abutaqa, Ziad F. Bulbul, Nour K. Younis, Mariam T. Arabi, Fadi F. Bitar

**Affiliations:** ^1^Department of Pediatrics and Adolescent Medicine, American University of Beirut Medical Center (AUBMC), Beirut, Lebanon; ^2^Department of Surgery, American University of Beirut Medical Center, Beirut, Lebanon; ^3^Faculty of Medicine, American University of Beirut Medical Center, Beirut, Lebanon

## Abstract

**Background:**

Aortic valve stenosis accounts for 3–6% of congenital heart disease. Balloon aortic valvuloplasty (BAV) is the preferred therapeutic intervention in many centers. However, most of the reported data are from developed countries.

**Materials and Methods:**

We performed a retrospective single-center study involving consecutive eligible neonates and infants with congenital aortic stenosis admitted for percutaneous BAV between January 2005 and January 2016 to our tertiary center. We evaluated the short- and mid-term outcomes associated with the use of BAV as a treatment for congenital aortic stenosis (CAS) at a tertiary center in a developing country. Similarly, we compared these outcomes to those reported in developed countries.

**Results:**

During the study period, a total of thirty patients, newborns (*n* = 15) and infants/children (*n* = 15), underwent BAV. Left ventricular systolic dysfunction was present in 56% of the patients. Isolated AS was present in 19 patients (63%). Associated anomalies were present in 11 patients (37%): seven (21%) had coarctation of the aorta, two (6%) had restrictive ventricular septal defects, one had mild Ebstein anomaly, one had Shone's syndrome, and one had cleft mitral valve. BAV was not associated with perioperative or immediate postoperative mortality. Immediately following the valvuloplasty, a more than mild aortic regurgitation was noted only in two patients (7%). A none-to-mild aortic regurgitation was noted in the remaining 93%. One patient died three months after the procedure. At a mean follow-up of 7 years, twenty patients (69%) had more than mild aortic regurgitation, and four patients (13%) required surgical intervention. Kaplan–Meier freedom from aortic valve reintervention was 97% at 1 year and 87% at 10 years of follow-up.

**Conclusion:**

Based on outcomes encountered at a tertiary center in a developing country, BAV is an effective and safe modality associated with low complication rates comparable to those reported in developed countries.

## 1. Introduction

Aortic valve stenosis represents around 3–6% of congenital heart disease, with an estimated incidence of 1–4 per 10,000 live births [1]. Aortic stenosis (AS) accounts for two-thirds of the lesions that cause obstruction of the left ventricular outflow tract. AS is likely an important contributing trigger to heart failure in neonates and infants. AS is particularly critical when the systemic circulation is mainly ductal dependent [2].

Currently, balloon aortic valvuloplasty (BAV) and surgical aortic valvotomy (SAV) are both employed in treating AS in neonates and infants. However, none of the interventions is considered the first-line treatment of AS [3]. Treatment choice depends on a multitude of factors including patient condition, physician experience, and hospital setting. Several studies aimed to assess the difference between BAV and SAV. Given this, SAV was found superior to BAV as per data reported from a few centers [4, 5]. Nevertheless, a recent meta-analysis by Hill et al. comparing the two interventions and involving 2,368 patients from 20 studies revealed no significant difference in long-term survival or freedom from aortic valve replacement between the two groups [6]. However, higher rates of reintervention were observed in the BAV group. Congruently, the IMPACT registry concluded that BAV is an effective treatment for congenital aortic stenosis (CAS) associated with low rates of mortality and adverse events [7].

Furthermore, most of the data regarding outcomes of BAV are obtained from studies performed in developed countries. Data regarding the experience and management of congenital aortic stenosis in developing countries are scarce [8–11].

The objective of our study is to evaluate the outcomes encountered at a tertiary cardiac center in a developing country. This is achieved through analysis of short- and mid-term results of BAV in neonates and children aged less than 2 years. Our study also aims to compare the outcomes observed in our country to those reported in developed countries.

## 2. Materials and Methods

### 2.1. Study Population

We included in our study all neonates and children younger than 2 years of age, who underwent BAV for CAS at the Children's Heart Center at the American University of Beirut-Medical Center (AUBMC) in Lebanon throughout a period of 11 years extending from January 2005 to January 2016 inclusive. All consecutive patients, satisfying the inclusion criteria, were enrolled in this retrospective single-center study. We excluded patients with associated subvalvular or supravalvular aortic stenosis, and patients deemed to have univentricular circulation.

### 2.2. Data Collection

Patients' data were collected from the AUBMC medical records, after securing institutional review board (IRB) approval. We studied the following parameters: patient demographics, general procedure characteristics, and hemodynamic findings obtained at baseline and on follow-up.

**Figure 1:**
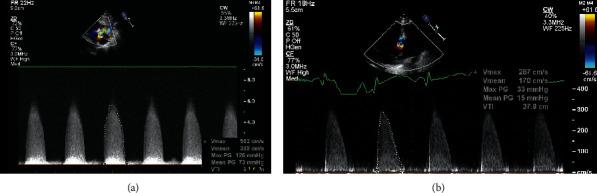
(a) An example of echocardiography preballoon angioplasty, continuous-wave spectral Doppler across the aortic valve in a newborn showing severe aortic stenosis with a mean gradient 73 mmHg and a peak systolic gradient 126 mmHg. (b). Echocardiography postballoon angioplasty, continuous-wave spectral Doppler across the aortic valve revealing marked decrease in the degree of aortic stenosis to a mean gradient of 15 mm Hg and a peak systolic gradient of 33 mmHg.

The aortic valve stenosis gradient was assessed by continuous and pulsed Doppler echocardiography from subcostal, apical, and right subclavicular approaches (see Figures 1(a) and 1(b)). The highest gradient measured was taken into account. The degree of aortic valve stenosis was classified as mild, moderate, or severe. This classification was based on aortic jet velocity and mean systolic gradient. AS was considered moderate to severe when the mean systolic gradient was above 50 mm Hg and the aortic jet velocity greater than 4 m/sec.

The morphology of the aortic valve was determined by two-dimensional (2D) echocardiography. Valves with thick leaflets were defined as dysplastic valves. The size of the aortic annulus was measured in the parasternal long axis view using echocardiography and confirmed by angiography. Radiographic modalities such as CT scan and MRI were not performed. Similarly, we used 2D echocardiography to measure the diameters and dimensions of the cardiac chambers during diastole and systole.

We inspected for the presence of both preprocedural and postprocedural aortic regurgitation (AR) using color flow mapping and continuous and pulsed Doppler. AR was categorized on a 4-grade scale as per the American Society of Echocardiography (ASE) recommendation. Here are the distinct grades of the ASE scale:

- No regurgitation
- Regurgitation without a reverse diastolic flow in the aortic arch (mild)
- Reversal flow in the aortic arch but not in the abdominal aorta (moderate)
- Reversal diastolic flow in the abdominal aorta (severe)

### 2.3. Transcatheter Balloon Aortic Valvuloplasty Procedure

**Figure 2:**
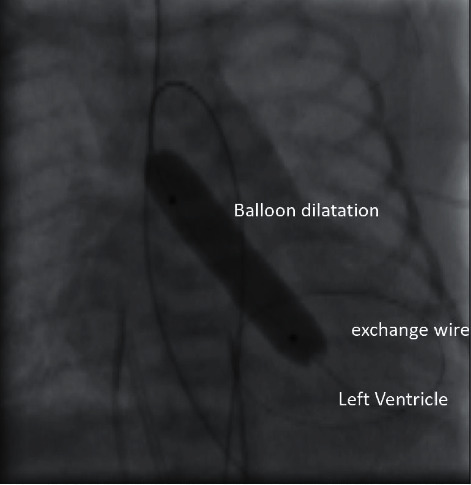
Selected cineradiographic frame showing a balloon positioned across the aortic valve for retrograde balloon valvuloplasty. The inflated balloon is placed over an exchange wire in the left ventricle. Additional nasogastric and umbilical venous and arterial catheters are seen.

The procedure was performed under general anesthesia, in the presence of the pediatric anesthesia team. The vascular access was secured through the arterial femoral approach and obtained percutaneously in all but one patient who necessitated arteriotomy through a surgical cut-down. Systemic unfractionated heparinization was administered at a dose of 50 IU/kg IV bolus to all patients. Valvuloplasty was performed using the transfemoral retrograde approach. The balloon diameter was often chosen equal to or slightly smaller than the aortic valve annulus. The type of balloon used was the Tyshak II Percutaneous Transluminal Valvuloplasty catheter (NuMED, Hopkinton, New York). The aortic valve was crossed retrograde using a Terumo 0.035-inch (”) guide wire and a 4 Fr (French) Judkins Right Coronary Catheter (JR) was placed in the left ventricle. A 0.018” wire was positioned in the LV apex. Meticulous manipulation was applied to avoid LV perforation or mitral valve injury. A low-pressure Nu MED Tyshak balloon catheter was advanced over the 0.018″ wire through the 4 Fr short sheath for the newborn patients (see Figure 2). A couple of hand inflations were performed during each procedure. However, we applied no rapid ventricular pacing during any of our procedures.

To note, balloon aortic valvuloplasty was indicated in all patients with moderate to severe aortic valve stenosis as assessed by 2D echocardiography. It was also offered to all newborns with critical aortic stenosis coupled with ductal dependent circulation or left ventricular dysfunction. In the latter case, BAV was provided regardless of the aforementioned echocardiographic parameters: aortic jet velocity and mean systolic gradient.

### 2.4. Assessment of Procedure Success

The procedure outcome was classified according to the degree of (1) gradient reduction reflected by the residual peak to peak systolic gradient recorded via cardiac catheterization and (2) aortic regurgitation at the end of the procedure. The following categories of outcomes were adopted from previously published data including the Multi-Center Safety and Efficacy Outcome Assessment study [7, 12, 13].

#### 2.4.1. Immediate Procedure Outcome

- *Optimal outcome*. Residual peak to peak systolic gradient (PSG) (by direct catheter measurement in catheterization laboratory) less than or equal to 35 mmHg, and trivial or no AR
- *Adequate outcome*. Residual peak to PSG less than or equal to 35 mmHg, and mild AR
- *Inadequate outcome*. Residual peak to PSG above 35 mmHg, and/or moderate or more AR

#### 2.4.2. Late Procedure Outcome

For comparison purposes and in order to maintain consistency at follow-up, we opted to define the late procedure success based upon the echocardiographic measurements obtained for the aortic valve.

- *Optimal outcome*. Less than mild residual stenosis and trivial or no AR (grade 0)
- *Adequate outcome*. Mild residual AS (defined by a Doppler peak instantaneous pressure gradient of equal to/less than 36 mm Hg) and mild AR (grade 1)
- *Inadequate outcome*. Moderate AS (mean gradient of 50 mm Hg by Doppler echocardiography) and/or moderate or more AR (grade 2)

In our analysis, we considered procedures with optimal or adequate outcomes successful.

### 2.5. Statistical Analysis

Data were presented as frequency, mean, and range. Statistical analysis was completed using SPSS (Statistical Package for the Social Sciences). A *p* value of less than 0.05 was considered statistically significant. Wilcoxon rank-sum test was used for continuous variables. The Kaplan–Meier estimate was used to obtain actuarial probabilities and to build the survival curve and the freedom from reintervention plot.

## 3. Results

### 3.1. Baseline Characteristics

In our study population, a total of 30 patients were included. The male-to-female ratio was 4 : 1. Neonates constituted half of the enrolled patients (*n* = 15). The mean age of the study population was 74 days (1 to 540 days) with a mean bodyweight of 4.5 kg (2 to 9.6 kg) (Table 1).

**Figure 3:**
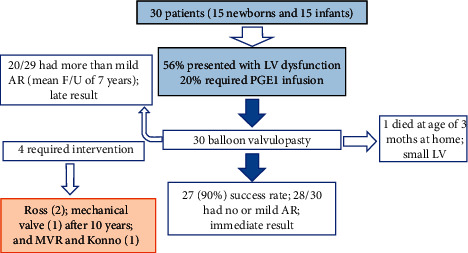
Outcomes of the studied population.

Left ventricle (LV) systolic dysfunction was a common finding (56%) with 20% of the patients requiring prostaglandin before the procedure (Figure 3). Isolated aortic valve disease was present in 61% of the study population. Associated anomalies were present in 37% of the patients: seven patients (23%) had coarctation of the aorta (CoA) (three were treated by surgery, three were treated by angioplasty, and one had trivial CoA), two (6%) had associated small restrictive ventricular septal defect (VSD), one had mild Ebstein anomaly, one had Shone's syndrome, and one had cleft mitral valve.

The mean size of the aortic annulus was 9 mm (4.7 to 11 mm). The mean aortic annulus diameter was 6.4 mm (4.7 to 8 mm) in newborns and 8.2 mm (6 to 11 mm) in infants. Forty percent of our patients had a bicuspid aortic valve while the remainder had a tricuspid valve (Table 1).

### 3.2. Transcatheter Balloon Aortic Valvuloplasty Procedure

There was almost complete agreement between the echocardiographic and angiographic measurements of the aortic annulus (*R* = 0.988). The resulting balloon-to-aortic annulus ratio ranged from 0.8 to 1.3 (mean ± SD; 1.03 ± 0.01). We started with a balloon/annulus ratio of 0.9–1. In the absence of an adequate response, the size of the balloon was gradually increased to achieve an acceptable response in the absence of a significant AR.

### 3.3. Immediate Results after Balloon Valvuloplasty

The average catheter peak to PSG decreased significantly from 78 mm Hg to 21 mm Hg (*p* < 0.001). The distribution of preintervention and postintervention peak AS gradients is summarized in Table 2. All but one patient had a residual catheter PSG of less than 35 mmHg after the procedure.

No hospital mortality was associated with the use of BAV in these patients. Major adverse peri-procedural complications occurred only in two newborns. One developed ventricular fibrillation upon inflation of the balloon; it was successfully cardioverted and he had no sequelae. The other newborn had a perforation of the right atrium (RA) that resulted in pericardial effusion that was recognized promptly and drained appropriately. The RA was sutured surgically with no hemodynamic compromise. RA perforation was induced by a pacing catheter used to reverse a transient heart block noted following the angioplasty.

The immediate aortic insufficiency after intervention, as assessed by echocardiography, was none to trivial in 12 of the patients (40%), mild aortic regurgitation in 16 patients (53%), and more than mild aortic regurgitation in 2 patients (7%) (Table 3).

Based on the aforementioned criteria, the procedure was considered successful in 90% of the performed cases, including both optimal and adequate results in 37% and 53%, respectively (Table 3).

### 3.4. Late Results after Balloon Valvuloplasty

The mean period of follow-up was 7 years (0.5 to 11 years). Only one patient passed away suddenly at home, three months after the procedure. This patient had a borderline small LV and Shone's complex.

On the last follow-up assessment by echocardiography, 70% of the patients had mild aortic stenosis, and 30% had more than mild AS: in 27% it was mild to moderate, and at least moderate in one patient (3%). As for aortic insufficiency, 14% (*n* = 4/29 alive) had none or trivial aortic regurgitation, 17% (*n* = 5) had mild aortic regurgitation, and 69% (*n* = 20) had more than mild AR.

**Figure 4:**
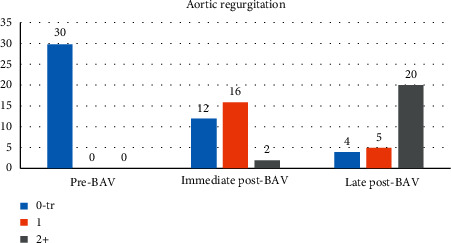
Distribution of the degree of aortic regurgitation (AR) before and after dilation among all patients (0: none to trivial AR, 1: mild AR, 2: more than mild AR).

Figure 4 depicts the progression in the degree of AR among the studied population.

### 3.5. Aortic Valve Reintervention

Four (13%) out of 30 patients required surgical interventions for the aortic valve (Table 4). One patient underwent mechanical valve replacement after 10 years from the procedure. The second had a Konno–Ross procedure 3 years after the valvuloplasty. The third underwent surgical aortic valve leaflet repair due to severe aortic insufficiency; he had resection of subaortic stenosis and repair of cleft mitral valve four years following the valvuloplasty. The fourth patient had a Ross procedure 4 years following the valvuloplasty due to severe AR.

Two out of the six patients with critical AS patients (33%) required late surgical intervention during the study period. In contrast, for the non-critical AS group consisting of 24 patients, two (8%) had late surgery.

**Figure 5:**
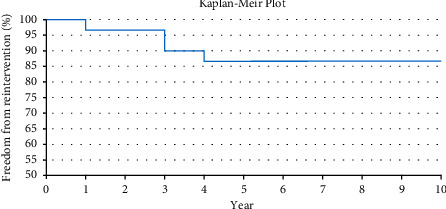
Graph showing the Kaplan–Meier survival free from aortic valve reintervention computed over a follow-up period of 10 years.

Kaplan–Meier freedom from aortic valve reintervention was 96.6% at 1 year, 90% at 3 years, and 86.6% at 10 years of follow-up (Figure 5). The actuarial survival probability in our study population was 0.97 (29/30) (Figure 3).

## 4. Discussion

The selection of the procedure of choice (BAV vs. SAV) is center specific. Randomized clinical trials are critically needed to assess the exact role of each procedure and also to endorse the superiority of one procedure over the other. BAV is the procedure of choice for the treatment of congenital aortic stenosis at our center. Immediate results from our study revealed a significant decrease in the peak to PSG of AS from 78 mm Hg prior to procedure to 21 mm Hg after intervention with only one patient (3.3%) having a residual PSG above 35 mm Hg. In a study reporting outcomes from 22 US centers, Torres et al. evaluated BAV as a treatment of AS in 373 patients [12]. More than three-quarters of the patients were infants and 85% of the patients had a residual catheter PSG ≤ 35 mm Hg after balloon aortic valvuloplasty. Overall, the success rate of BAV was estimated at 71%, and no procedural mortality was noted [12].

Immediately after dilatation, our study achieved 90% success in terms of optimal and adequate outcomes, with only 7% of patients having more than mild AR and 3% having a residual gradient of more than 35 mm Hg. The IMPACT study, the largest study of its kind from the USA, described 1,026 isolated BAV procedures [7]. 718 (70%) were reported to be “successful.” Success rates were 70.9% for noncritical AS (*n* = 916) and 62.7% for critical AS (*n* = 110). The IMPACT study revealed an in-hospital death of 1.5% for noncritical AS and 10.0% for critical AS as compared to 0% in-hospital mortality in our study for both critical and noncritical AS, and 3% mortality on follow-up. Based on data described from developed countries, particularly from UK, USA, and Canada, procedural mortality rates ranged between 2% and 4% in patients with noncritical AS [14–16], and between 9% and 14% in patients with critical AS [17–19].

Major adverse peri-procedural complications occurred in two newborns (7%) with critical AS in our study. The IMPACT study revealed major adverse events of 9.6% for the non-critical AS group and 27.3% for the critical AS group. Ewert et al. analyzed 1004 patients with AS who underwent BAV at 20 different German centers; 58% were newborns and infants [20]. Complications rates of 15% and 11% were noted in newborns and infants, respectively [20].

The immediate aortic insufficiency after intervention was none to trivial in 40%, mild in 53%, and more than mild in 7% of our patients. This is similar to what is reported in other series [17, 21]. In our study, the two patients who developed more than mild AR following the BAV were newborns with critical, ductal-dependent lesions and one of them had Shone's complex, with borderline small LV. Both patients had bicuspid aortic valves, with a relatively large balloon-to-aortic annulus ratio of 1.14 and 1.27, respectively, utilized for the valvuloplasty. However, the overall balloon-to-aortic annulus ratio used in our study was between 0.8 and 1.3, with a mean of 1.

In our study, we did not use ventricular pacing during BAV in any of our patients. In fact, balloon positioning can be achieved in newborns and infants with no significant difficulties if long balloons are used. However, rapid ventricular pacing may be mandatory for optimal balloon positioning during BAV in children, adolescents, and adults. Rapid ventricular pacing can be achieved through (1) direct LV guidewire pacing or (2) regular pacing mediated by a temporary pacemaker (PM) placed in the right ventricle [22].

The potential late complications of BAV are reported in various studies [23, 24]. They include primarily residual re-stenosis, worsening aortic regurgitation, and need for surgical intervention or AVR. In our study, the degree of aortic regurgitation progressed during the course of follow-up, and the reintervention rate after balloon valvuloplasty was 13% during the mid-term follow-up period. Four patients had surgical intervention: one had mechanical valve replacement due to severe AR, one patient had Konno–Ross due to LV outflow tract obstruction and severe AR, one had aortic valve repair because of severe AR with resection of subaortic stenosis, and one had Ross procedure due to severe AR. Kaplan–Meier survival free from aortic valve reintervention was 87% at 10 years of follow-up in our study with an actuarial survival probability of 0.97.

In a retrospective study involving 154 patients with CAS who received BAV at Seattle Children's Hospital in Washington, Sullivan et al. reported that 11% of patients who underwent neonatal BAV and 58% of those who underwent intervention past the neonatal period remained free from moderate-severe AR at a follow-up of 15 years after BAV [24]. In our study, only 31% were free from moderate-severe AR at a mean follow-up of 7 years.

Similarly, Pedra and colleagues reported the late outcomes after aortic valve dilation in 87 children over a mean period of 6.3 years and noted a 67% and 46% freedom from aortic valve reintervention at 5 and 12 years, respectively [25]. The reintervention rate was found to be higher in newborns who received valve dilatation for critical AS [25]. In our cohort, out of the six patients with critical AS, two patients (33%) required late surgical intervention. However, for the non-critical AS group, only two of 24 patients (8%) had late surgery (Table 4).

Studies from developing countries regarding the management of congenital aortic stenosis are scarce [9]. Data regarding balloon valvuloplasty for congenital aortic stenosis in developing countries are limited. Rossi et al. from Brazil reported that percutaneous intervention for relief of critical aortic stenosis in newborns in a developing country is safe and has results comparable to those reported in other centers throughout the world [8]. Congruently, Jindal et al. and Awasthy et al. from India reported that favorable outcomes are associated with the use of BAV as a treatment for CAS [10, 11].

## 5. Study Limitations

The main limitation of our study is inherent to the retrospective study design, and the small number of studied patients. The small size of our study population hindered us from analyzing the independent predictors of outcomes by logistic regression. Additionally, the immediate procedure outcomes were assessed based on the degree of gradient reduction reflected by the residual peak to peak systolic gradient and the degree of aortic regurgitation at the end of the procedure recorded via cardiac catheterization. However, the late procedure outcomes were based on the echocardiographic measurements of the aortic valve. This can affect the assessment of the short- and mid-term outcomes of the procedure and may result in small discrepancies.

## 6. Conclusion

BAV is a safe and effective modality to treat CAS in newborns and infants, even in the presence of additional anomalies (i.e., CoA). Early and late mortality rates are low. A significant progression in the degree of aortic regurgitation after valvuloplasty was noted during the course of follow-up; however, the rate of aortic reintervention remained relatively low. Ultimately, we conclude that favorable outcomes are associated with the use of BAV in a tertiary center in a developing country. These outcomes are comparable to those reported in developed countries.

## Figures and Tables

**Table 1:** Baseline characteristics of enrolled patients with congenital aortic stenosis.

	Newborns (*n* = 15)	Infants (*n* = 15)
Mean bodyweight (kg)	3.6 (2–7)	5.3 (2.9–9.6)
Mean aortic annulus diameter (mm)	6.4 (4.7–8)	8.2 (6–11)

*Aortic valve anatomy*		
Bicuspid	6 (40%)	6 (40%)
Tricuspid	9 (60%)	9 (60%)

**Table 2:** Summary of the distribution of preintervention and postintervention peak systolic gradient (PSG) measured during cardiac catheterization.

Peak AS gradient (mmHg)	Number (%)
*Preintervention peak AS gradient*	
≤49	2 (6.6%)
50–79^*∗*^	16 (53.4%)
≥80	12 (40%)

*Immediate postintervention peak AS gradient*	
≤35	29 (96.6%)
≥35	1 (3.3%)

^*∗*^Some gradients were underestimated due to the presence of LV dysfunction at the time of presentation.

**Table 3:** Distribution of patients as per aortic regurgitation (AR) severity and outcome category immediately after balloon aortic valvuloplasty.

Variable	Value *n* (%)
*Immediate postdilation AR severity*	
None to trivial	12 (40%)
Mild	16 (53%)
More than mild	2 (7%)

*Outcome category*	
Optimal	11 (37%)
Adequate	16 (53%)
Inadequate	3 (10%)

**Table 4:** Percentage of patients who received late surgical intervention and % of freedom from aortic valve reintervention at 10 years of follow-up in the groups of critical and noncritical AS, respectively.

AS groups	Number of patients	Number of patients who received late surgical intervention	% of patients who received late surgical intervention (%)	% of freedom from aortic valve reintervention at 10 years of follow-up (%)
Critical AS group	6	2	33	67
Non-critical AS group	24	2	8	92
Overall	30	4	13	87

Abbreviations: AS: aortic stenosis.

## Data Availability

The data are available from the corresponding authors (MTA and FFB) upon reasonable request.

## References

[1] Khalid O., Luxenberg D. M., Sable C. (2006). Aortic stenosis: the spectrum of practice. *Pediatric Cardiology*.

[2] Magee A. G., Nykanen D., McCrindle B. W., Wax D., Freedom R. M., Benson L. N. (1997). Balloon dilation of severe aortic stenosis in the neonate: comparison of anterograde and retrograde catheter approaches. *Journal of the American College of Cardiology*.

[3] Benson L. (2016). Neonatal aortic stenosis is a surgical disease: an interventional cardiologist view. *Seminars in Thoracic and Cardiovascular Surgery: Pediatric Cardiac Surgery Annual*.

[4] Bhabra M. S., Dhillon R., Bhudia S. (2003). Surgical aortic valvotomy in infancy: impact of leaflet morphology on long-term outcomes. *The Annals of Thoracic Surgery*.

[5] Hraska V., Sinzobahamvya N., Haun C. (2012). The long-term outcome of open valvotomy for critical aortic stenosis in neonates. *The Annals of Thoracic Surgery*.

[6] Hill G. D., Ginde S., Rios R., Frommelt P. C., Hill K. D. (2016). Surgical valvotomy versus balloon valvuloplasty for congenital aortic valve stenosis: a systematic review and meta-analysis. *Journal of the American Heart Association*.

[7] Boe B. A., Zampi J. D., Kennedy K. F. (2017). Acute success of balloon aortic valvuloplasty in the current era. *JACC: Cardiovascular Interventions*.

[8] Rossi R. I., Manica J. L. L., Petraco R., Scott M., Piazza L., Machado P. M. (2011). Balloon aortic valvuloplasty for congenital aortic stenosis using the femoral and the carotid artery approach: a 16-year experience from a single center. *Catheterization and Cardiovascular Interventions*.

[9] Al-Halees Z., Pieters F., Qadoura F., Shahid M., Al-Amri M., Al-Fadley F. (2002). The Ross procedure is the procedure of choice for congenital aortic valve disease. *The Journal of Thoracic and Cardiovascular Surgery*.

[10] Jindal R. C., Saxena A., Juneja R., Kothari S. S., Shrivastava S. (2000). Long-term results of balloon aortic valvulotomy for congenital aortic stenosis in children and adolescents. *The Journal of Heart Valve Disease*.

[11] Awasthy N., Garg R., Radhakrishnan S., Shrivastava S. (2016). Long-term results of percutaneous balloon valvuloplasty of congenital aortic stenosis in adolescents and young adults. *Indian Heart Journal*.

[12] Torres A., Vincent J. A., Everett A. (2015). Balloon valvuloplasty for congenital aortic stenosis: Multi-center safety and efficacy outcome assessment. *Catheterization and Cardiovascular Interventions*.

[13] Porras D., Brown D. W., Rathod R. (2014). Acute outcomes after introduction of a standardized clinical assessment and management plan (SCAMP) for balloon aortic valvuloplasty in congenital aortic stenosis. *Congenital Heart Disease*.

[14] Rocchini A. P., Beekman R. H., Shachar G. B., Benson L., Schwartz D., Kan J. S. (1990). Balloon aortic valvuloplasty: results of the valvuloplasty and angioplasty of congenital anomalies registry. *The American Journal of Cardiology*.

[15] McCrindle B. W. (1996). Independent predictors of immediate results of percutaneous balloon aortic valvotomy in childhood. *The American Journal of Cardiology*.

[16] Sholler G. F., Keane J. F., Perry S. B., Sanders S. P., Lock J. E. (1988). Balloon dilation of congenital aortic valve stenosis. Results and influence of technical and morphological features on outcome. *Circulation*.

[17] McElhinney D. B., Lock J. E., Keane J. F., Moran A. M., Colan S. D. (2005). Left heart growth, function, and reintervention after balloon aortic valvuloplasty for neonatal aortic stenosis. *Circulation*.

[18] Egito E. S. T., Moore P., O’Sullivan J. (1997). Transvascular balloon dilation for neonatal critical aortic stenosis: early and midterm results. *Journal of the American College of Cardiology*.

[19] Hamidi-Manesh L., Tibby S. M., Herman R., Rosenthal E., Qureshi S. A., Krasemann T. (2013). Influence of balloon size on aortic regurgitation in neonates undergoing balloon aortic valvuloplasty-A retrospective study over an 11-year period. *Journal of Interventional Cardiology*.

[20] Ewert P., Bertram H., Breuer J. (2011). Balloon valvuloplasty in the treatment of congenital aortic valve stenosis - a retrospective multicenter survey of more than 1000 patients. *International Journal of Cardiology*.

[21] Moore P., Egito E., Mowrey H., Perry S. B., Lock J. E., Keane J. F. (1996). Midterm results of balloon dilation of congenital aortic stenosis: predictors of success. *Journal of the American College of Cardiology*.

[22] Kleczynski P., Dziewierz A., Socha S. (2020). Direct rapid left ventricular wire pacing during balloon aortic valvuloplasty. *Journal of Clinical Medicine*.

[23] Reich O., Tax P., Marek J. (2004). Long term results of percutaneous balloon valvoplasty of congenital aortic stenosis: independent predictors of outcome. *Heart*.

[24] Sullivan P. M., Rubio A. E., Johnston T. A., Jones T. K. (2017). Long-term outcomes and re-interventions following balloon aortic valvuloplasty in pediatric patients with congenital aortic stenosis: a single-center study. *Catheterization and Cardiovascular Interventions*.

[25] Pedra C. A. C., Sidhu R., McCrindle B. W. (2004). Outcomes after balloon dilation of congenital aortic stenosis in children and adolescents. *Cardiology in the Young*.

